# RNA Interference Technology to Control Pest Sea Lampreys - A Proof-of-Concept

**DOI:** 10.1371/journal.pone.0088387

**Published:** 2014-02-05

**Authors:** George Heath, Darcy Childs, Margaret F. Docker, David W. McCauley, Steven Whyard

**Affiliations:** 1 Department of Biological Sciences, University of Manitoba, Winnipeg, Manitoba, Canada; 2 Department of Biology, University of Oklahoma, Norman, Oklahoma, United States of America; Natural Resources Canada, Canada

## Abstract

The parasitic sea lamprey (*Petromyzon marinus*) has caused extensive losses to commercial fish stocks of the upper Great Lakes of North America. Methods of controlling the sea lamprey include trapping, barriers to prevent migration, and use of a chemical lampricide (3-trifluoromethyl-4-nitrophenol) to kill the filter-feeding larvae. Concerns about the non-specificity of these methods have prompted continued development of species-specific methods to control lampreys outside their native range. In this study, we considered the utility of RNA interference to develop a sea lamprey-specific lampricide. Injection of six different short interfering, double-stranded RNAs (siRNAs) into lamprey embryos first confirmed that the siRNAs could reduce the targeted transcript levels by more than 50%. Two size classes of lamprey larvae were then fed the siRNAs complexed with liposomes, and three of the siRNAs (targeting elongation factor 1α, calmodulin, and α-actinin) reduced transcript levels 2.5, 3.6, and 5.0–fold, respectively, within the lamprey midsections. This is not only the first demonstration of RNAi in lampreys, but it is also the first example of delivery of siRNAs to a non-mammalian vertebrate through feeding formulations. One of the siRNA treatments also caused increased mortality of the larvae following a single feeding of siRNAs, which suggests that prolonged or multiple feedings of siRNAs could be used to kill filter-feeding larvae within streams, following development of a slow-release formulation. The genes targeted in this study are highly conserved across many species, and only serve as a proof-of-concept demonstration that siRNAs can be used in lampreys. Given that RNA interference is a sequence-specific phenomenon, it should be possible to design siRNAs that selectively target gene sequences that are unique to sea lampreys, and thus develop a technology to control these pests without adversely affecting non-target species.

## Introduction

Since its arrival into the upper Great Lakes of North America, the parasitic sea lamprey (*Petromyzon marinus*) has caused extensive losses to commercial fish stocks. By the 1950s, the commercial fishing industries were suffering from devastating losses as sea lampreys were depleting fish stocks [1]. To deal with this serious invasive species, the Great Lakes Fishery Commission (GLFC) sea lamprey control program was initiated. Management initially relied primarily on stream treatments with a selective lampricide (3-trifluoromethyl-4-nitrophenol, TFM) to kill sea lamprey larvae, on barriers to prevent migration, and on trapping to remove potential spawners [2]. Although sea lamprey abundance has been reduced by 90% from peak levels and is considered the only reported successful control program for a non-native, vertebrate pest species [3], there are growing concerns about the continued use of chemical lampricides and their potential negative impacts on juvenile lake sturgeon [4]–[6] and native lamprey species [7],[8].

As a result of these concerns, sea lamprey-specific control measures have been sought. For example, a sterile-male-release technique (SMRT) for sea lamprey was developed in the 1970s [9] and, starting in 1991, was used in some Great Lakes tributaries as part of an integrated pest management strategy [10], [11]. However, the SMRT was suspended in 2011; the potential human health hazards of the chemosterilant, bisazir, restricted its use to a single specialized facility in Michigan [10] and this, combined with its cost and the low trapping success of males for sterilization, limited the number of sterile males that could be released to compete with wild fertile males [12].

Other alternative control strategies that are being pursued include the use of pheromones to disrupt sea lamprey migration [13],[14] or reproduction [15]. These pheromones could potentially be used to enhance trapping or to divert migrating or spawning lampreys to areas with poor larval habitat or away from areas that are difficult to treat with lampricides [16],[17]. Field trials to date have been promising [4],[13], but additional research is required to optimize strategies that will be practical, effective, and economical [14],[17], as well as environmentally benign. The migratory pheromone, for example, may also be used by native lamprey species [18] and its species specificity has yet to be fully explored.

Increased species specificity of the aforementioned technologies or the development of additional sea lamprey control methods could be achieved using newly acquired genetic information. In 2004, the sea lamprey genome sequencing project was started, and with its recent completion [19], it is now possible to look for unique genes or sequences that could lead to lamprey-specific control methods. To harness the genomic information for sea lamprey control purposes, we must be able to identify those sequences that are sea lamprey-specific and to develop technologies that target those unique features. Sequence comparisons with other genomes will allow us to predict the function of numerous genes, but the functions of the majority of genes will remain unknown until they can be experimentally tested.

There is a growing repertoire of molecular biology tools that have been developed to determine the function of genes, but one of the most versatile of these is RNA interference (RNAi). RNAi is a gene-silencing technique that uses double-stranded RNA (dsRNA) to induce the destruction of any RNA molecule with sequence identity to the dsRNA, thereby reducing or eliminating the target gene's expression. This sequence-specific gene silencing effect has been widely used in molecular biology studies to create loss-of-expression phenotypes that have elucidated the role of various genes in numerous eukaryotic organisms [20]. To date, RNAi has not been applied in lampreys, but given the ease with which it has been used in many other organisms, its application in lampreys would prove useful in validating the function of the newly-identified genes, particularly if an efficient method of dsRNA delivery could be achieved.

In addition to its utility as a gene validation tool, RNAi has considerable potential for a range of applications, including the development of pest control technologies. Numerous examples of inducing RNAi simply by feeding dsRNA to various invertebrates have been described [21]. Two research groups demonstrated that plants could be genetically engineered to express insect-specific dsRNAs that would kill the insects that feed on them [22],[23]. Given that RNAi operates in a very sequence-specific manner, it may be possible to develop species-specific dsRNA pesticides. Even when a highly conserved gene such as γ-tubulin is targeted by RNAi, dsRNAs specific to the unique 3′ untranslated region (3′ UTR) of the mRNAs could selectively kill only one species of *Drosophila*, without adversely affecting other closely related drosophilids [24].

Based on these intriguing potential applications of RNAi in invertebrates, this study sought to establish whether RNAi could be applied to lampreys, both for use as a gene validation tool to identify the functions of newly-identified lamprey genes, and as a biotechnological approach to control sea lamprey larvae, without adversely affecting non-target species that share their habitat. We provide evidence that RNAi can be induced in both sea lamprey embryos and feeding larvae, and that at least one siRNA tested could prevent their development. These results serve as a proof-of-concept that siRNAs, given the sequence-specificity of RNAi, have the potential for use as sea lamprey-specific lampricides.

## Materials and Methods

### Ethics Statement

This study was carried out in strict accordance with the recommendations in the Guidelines of the Canadian Council on Animal Care and the Guide for the Care and Use of Laboratory Animals of the National Institutes of Health. The protocol was approved by the Protocol Management and Review Committee of the University of Manitoba (Permit Number: F09-028). Collection of lampreys in Manitoba was approved by the Manitoba Water Stewardship – Aquatic Ecosystem Section (Permit number 05-11) and collection of sea lampreys was approved by the State of Michigan, Scientific Collector's Permit issued under the provisions of Part 487, Act 451, P.A. 1994 to Hammond Bay Biological Station. Transport of sea lampreys to the University of Oklahoma was approved by the State of Oklahoma, Permit to Import/Export Aquatic Wildlife (Permit numbers 2010-370 and 2011-008).

### Animal Collection and Maintenance

Sexually mature adult sea lampreys were collected from live traps in the Ocqueoc River (MI, USA) during the month of June. Gametes were manually stripped from the adults and used to fertilize the eggs, as previously described [25],[26]. The developing embryos were maintained at 18°C in a circulating water system as described by Piavis (1961) [27], until needed for siRNA injections or for RNA extractions.

Young-of-the-year (YOY) sea lamprey larvae were collected from the Trout River (MI, USA) during early August, when the animals were approximately 10–12 weeks old and ranged in size from 10 to 30 mm in length. The larvae were maintained in 500 ml glass bowls sealed with a screen-covered lid, with continuous flow of Lake Huron water. Older larvae (1 yr+), ranging in length from 40 to 80 mm, were provided by the Hammond Bay Biological Station (MI, USA) lamprey pheromone farm. These lampreys had been collected from the Trout River the previous year, and had been maintained in large totes (∼100 L) containing Lake Huron water and 3-10 cm of sand to permit larval burrowing. Both YOY and 1 yr+ larvae were used in siRNA feeding bioassays (described below).

To infer species-specificity, small numbers (<15) of northern brook lamprey larvae (*Ichthyomyzon fossor*) were collected from the Birch River (near Winnipeg MB, Canada); these lampreys represent one of the four native lamprey species found in the Great Lakes basin. They were euthanized with an overdose of tricaine methanesulfonate (MS-222) and were sectioned into anterior, middle, and posterior sections. The sections were snap frozen in liquid nitrogen and stored at −80°C until needed for RNA extractions.

### siRNA Target Genes

Six housekeeping genes were selected as targets for siRNA design: β actin, α actinin, calmodulin, elongation factor 1α, splicing factor 1 and γ tubulin. Putative homologues of either zebrafish (*Danio rerio*) or Arctic lamprey (*Lethenteron camtschaticum*) genes were identified within the sea lamprey genome (http://www.ensembl.org/Petromyzon_marinus/blastview) using BLAST, and the primers designed to PCR-amplify the sea lamprey genes are listed in Table 1.

**Table 1:** PCR primers used to amplify target gene fragments from sea lamprey cDNA.

Target gene	Sea lamprey Contig	Primer sequences	Predicted PCR amplicon length
Actin	GL498954	F: CCATCCAGGCCGTGCTGTCG R: AGGACGGCTGGAAGAGGGCC	353 bp
Actinin	GL479988	F: ATACTTGAGCTCCTGGGCA R: AGGGCAAGATGGTCTCGG	289 bp
Calmodulin	GL476713	F: TGCAGAGTTCAAGGAGGGCG R: TGAACATACCATCGGCATCC	119 bp
Elongation factor	GL499053	F: CACGTTCAACTGCAATGTTTAT R: TTCCAGGGCGCTTGCCGT	332 bp
Splicing factor	GL478966	F: CAGGAACGGCCCGGAGTT R: CATCTGAAAGCTCCGCGATG	251 bp
Tubulin	GL476764	F: TTTTAACTAGGTGTCGACTAT R: ATCAGTACCAGGCATATTTAAC	269 bp

F =  forward primer, R =  reverse primer.

RNA was isolated from 20 pooled sea lamprey embryos and from 3 pooled midsections of YOY or 1 yr+ larvae using an RNeasy Kit and Qiashredder (Qiagen). The RNA was treated with amplification grade DNase I (Invitrogen) and 1 µg RNA was then used to synthesize cDNA using a QuantiTect Reverse Transcription Kit (Qiagen) with random hexamers. To confirm that each of the target genes was expressed, the cDNA was used as template to PCR-amplify portions of each gene, using primers listed in Table 1. The PCR products were resolved on agarose gels and were sequenced (Robarts Sequencing Facility, London, ON, Canada) to confirm their identity.

### siRNA Delivery for Sea Lamprey Embryos and Larvae

Synthetic Stealth™ siRNAs (Invitrogen) for each of the target genes and for a negative control (targeting the *E. coli* gene encoding β-glucuronidase, *gus*) were designed using the BLOCK-iT™ RNAi Designer software (Table 2).

**Table 2:** Sea lamprey target gene contigs and associated siRNA sequences.

Target gene	Stealth siRNA sequence
α-actinin	CAAUGGACACGAGUUUCACACCCUU
Actin	GCAAGCGUGGUAUCCUCACCCUUAA
Calmodulin	CAUGAUCAAUGAGGUGGAUGCCGAU
Elongation Factor 1a	UGAACGUCACCACUGAGGUCAAGUC
Splicing Factor 3a	CAGCACAGCCUCUUCCACUACUUCA
Tubulin	CAGGCUACAUGAACAAUGACCUCAU
Gus^a^	GGAUCAACAGGUGGUUGCAACUGGA

a NCBI accession # for the *E. coli* β glucuronidase (*gus*) gene  = A00196.1

Three groups of ∼100 fertilized embryos were injected with Stealth RNAi (Invitrogen) siRNAs specific to each of the six sea lamprey genes or the negative control β-glucuronidase gene of *Escherichia coli*. One or two-cell stage embryos were injected with ∼10 nl of each siRNA at 20 µM concentration (dissolved in water) using a Nanoject single line microinjector. Embryos were reared in a recirculating water system at 18°C according to Piavis (1961) [27]. Embryos were monitored for three days to assess whether they developed normally, beyond the blastula stage. RNA was extracted from 10–30 embryos on day 3 post-injection, using an RNeasy Kit and Qiashredder (Qiagen) and stored at −80°C.

To deliver siRNAs to feeding stage lamprey larvae, Stealth RNAi (Invitrogen) siRNAs (50 µl of 20 µM stock) were individually mixed with either 10 µl Lipofectamine™ (Invitrogen) or water, and after 5 min incubation at room temperature, the siRNA mixtures or Lipofectamine alone were added to 15 ml plastic tubes (Falcon) containing 5 ml deionized water, 25 young-of-the- year (YOY) sea lampreys (ranging in length from 15 to 20 mm long) and 500 ml of a yeast feeding suspension (100 mg/ml). The larvae were exposed to the siRNAs for 6 h at 18°C. To assess whether the larvae would feed in the siRNA treatment test tubes, a pilot experiment was performed with blue food coloring mixed with the siRNA/yeast suspension. As all larvae had some food coloring visible within their guts, the dye was not used thereafter. Following exposure to the siRNA mixture, the larvae were transferred to glass bowls and allowed to burrow into sand. At 48 and 72 hours post treatment, five animals were anesthetized with an overdose of MS-222 and crudely dissected to isolate head, mid-section and tail regions. RNA was extracted from the dissected tissues as described above and stored at −80°C. The remaining YOY were observed daily until day 10 post-treatment to assess any effects on the viability of the larvae over time.

Older (1 yr+) larvae were individually placed in 15 ml Falcon tubes containing 10 ml deionized water, 20 µl Lipofectamine (Invitrogen), 100 µl Stealth RNAi (Invitrogen) and 500 µl of yeast. The larvae were treated in a similar manner as the YOY larvae, except that at 72 h post-treatment, the animals were exposed to an overdose of MS-222 and gut and liver tissues were dissected. Total RNA was extracted as previously described and stored at −80°C.

### QRT-PCR Assessment of RNAi

The extent of RNAi was determined using quantitative RT-PCR (qRT-PCR). RNA derived from the embryos was first treated with amplification grade DNase I (Invitrogen) and 1 µg RNA was then used to synthesize cDNA using a QuantiTect Reverse Transcription Kit (Qiagen) with random hexamers. The cDNA was used as template in qRT-PCR reactions performed in triplicate on a BioRad iQ5 Real-Time PCR Detection System using the primers listed in Table 3. Note that the qRT-PCR primers were designed to amplify portions of the genes not targeted by the siRNAs. A gene with high sequence identity to the zebrafish S7 ribosomal protein gene (NCBI accession AY561508.1) was used as an internal reference to compare relative levels of gene expression. A single reference gene was deemed sufficient, as the PCR efficiencies of the primer sets were calculated using the method of Pfaffl (2001) [28], and were found to be essentially equivalent for all genes examined, with values ranging between 95.0 and 97.5%. Melt curve analyses were also performed and confirmed that only a single product was amplified with each primer pair in every sample. Analysis of gene expression was performed using the 2^−ΔΔC^_T_ method [29], and the values are expressed as relative gene expression, by comparing expression in gene-specific siRNA-treated samples to *gus*-siRNA-treated samples.

**Table 3:** Primers used for qRT-PCR.

Gene	Reverse primer	Forward primer
α-Actinin	AGGGCAAGATGGTCTCGG	ATACTTGAGCTCCTGGGCA
Actin	TCGCTACAGGCAGCATCCC	CACAGATCATGTTCGAGACC
Calmodulin	TGAACATACCATCGGCATCC	TGCAGAGTTCAAGGAGGCG
Elongation Factor	TTCCAGGGCGCTTGCCGT	CACGTTCAACTGCAATGTTTATT
Splicing Factor	CATCTGAAAGCTCCGCGATG	CAGGAACGGCCCGGAGTT
Tubulin	ATCAGTACCAGGCATATTTAAC	TTTTAAACTAGGTGTCGACTATC
Ribosomal S7	AACTGGGACAAAGATAAGG	TTGGAACTGGAGATGAAC

### Data Analysis

All statistical analyses were calculated using SPSS for Windows (SPSS, Chicago, IL, USA). The relative mRNA transcript level for each individual lamprey (siRNA-treated and controls) was calculated from three experimental replicate qRT-PCR measurements, and displayed using scatter plots. The median gene expression level for each group of treatments was then calculated and compared to the control treatments using Mann-Whitney U tests. The mortalities of siRNA-treated embryos were compared to that of negative control larvae that were treated with the *E.coli*-specific *gus*-siRNA using student t-tests. Mortalities of larvae treated with lamprey-siRNAs were compared to that of the *gus*-siRNA treated larvae using Chi-square (χ^2^) analyses with Yates' correction for continuity, as these analyses were based on smaller sample sizes (two replicates of 15 individuals for each treatment).

## Results

### Choice of Genes to Assess RNAi in Sea Lampreys

As RNAi had not previously been demonstrated in sea lampreys, the first goal of this study was to assess whether RNAi could be induced in different early life stages of sea lampreys. Six candidate genes (β-actin, α-actinin, calmodulin, elongation factor1α, splicing factor 1, and γ-tubulin) were selected as RNAi targets with the expectation that at least one of them would have a measureable effect on the early life stages of the sea lampreys. All of the genes chosen are highly conserved across a range of taxa, which facilitated their identification from the sea lamprey genome database. Sequence alignments of the nucleotide sequences using CLUSTALW found that all six genes shared over 75% identity with their respective zebrafish homologues (Table 4). RT-PCR successfully amplified each of the target genes from cDNA derived from both embryos and larval tissues (results not shown), indicating that the identified genes were expressed during these stages of lamprey development.

**Table 4:** Nucleotide sequence identities of the target gene fragments in sea lamprey and zebrafish.

Gene	Sea lamprey contig	Zebrafish NCBI accession	% identity (length of gene fragment)
Actin	GL498954	NM131031.1	98% (353 bp)
Actinin	GL479988	AF524840.1	84% (289 bp)
Calmodulin	GL476713	BC053150.1	99% (119 bp)
Elongation factor	GL499053	AB183717.1	99% (332 bp)
Splicing factor	GL478966	BC163938.1	75% (251 bp)
Tubulin	GL476764	BC159223.1	89% (269 bp)

### Injected siRNAs Induced RNAi and Mortality in Sea Lamprey Embryos

To determine if the siRNAs could induce RNAi in sea lampreys, one- or two-celled sea lamprey embryos were injected with each of the siRNAs. By 3 days post-treatment, the survival of the embryos was assessed by determining whether development had ceased (by assessing whether development had progressed to or beyond the blastula stage). RNA was then extracted from the embryos and qRT-PCR analysis revealed that of the six siRNAs tested, four (actinin, calmodulin, splicing factor, and tubulin) showed almost 50% or more knockdown of the target gene expression in the embryos, relative to the *gus*-siRNA-treated controls (Table 5). Two siRNAs, designed to target actin and elongation factor 1α mRNAs, failed to induce significant knockdown of their respective transcripts.

**Table 5:** Induced mortality and extent of RNAi in sea lamprey embryos following siRNA injections.

Injection treatment	% embryo mortality*	% RNAi
*gus*-siRNA (negative control)	8.9±5.5	0±4
*actin*-siRNA	15.5±5.1	5±3
*actinin*-siRNA	23.5±4.3 ^a^	68±6 ^b^
*calmodulin*-siRNA	29.5±12.6 ^a^	49±6 ^b^
*elongation factor-1α* siRNA	9.8±3.3	10±3
*splicing factor*-siRNA	40.5±17.4 ^a^	86±6 ^b^
*tubulin*-siRNA	11.0±4.0	53±6 ^b^

* The % mortality values represent the means and standard errors for 6 experiments (of at least 30 embryos). The extent of RNAi is based on three replicates of 20 pooled embryos and is expressed relative to the *gus*-siRNA negative controls.

a – significantly greater mortality than the *gus*-siRNA negative controls (student t-test, P<0.05)

b – significantly different than the *gus*-siRNA negative controls (student t-test, P<0.05).

Three of the six siRNAs (actinin, calmodulin and splicing factor) not only induced a relatively strong RNAi response in the embryos, but they also induced a significantly higher degree of mortality in the embryos, relative to the *gus*-siRNA-injected negative controls (Table 5; P<0.05, student t-test). The *gus*-siRNA appeared to have no adverse effects on the embryos, as only 8.9±3.1% of the *gus*-siRNA injectants failed to develop to the blastula stage by day 3, while a comparable 8.4±3.1% of non-injected embryos also failed to develop (i.e. no significant difference, P>0.5, student t-test). Actinin and calmodulin siRNAs caused an increase in embryo mortality of almost 15 and 20%, respectively, relative to the negative control embryos. The splicing factor siRNA had the most potent effect on the embryos, causing about 30% higher mortality in the embryos, relative to the negative controls. Interestingly, the tubulin-siRNA induced a moderately high level of RNAi (53%, P<0.05, student t-test) relative to the negative controls, but this siRNA did not induce a corresponding increase in mortality. In contrast, the calmodulin siRNA induced a slightly weaker RNAi response (49%, P<0.05, student t-test), but this was sufficient to induce a modest, yet significant, increase in mortality.

### Oral Delivery of siRNAs Induced RNAi in Sea Lamprey Larvae

**Figure 1:**
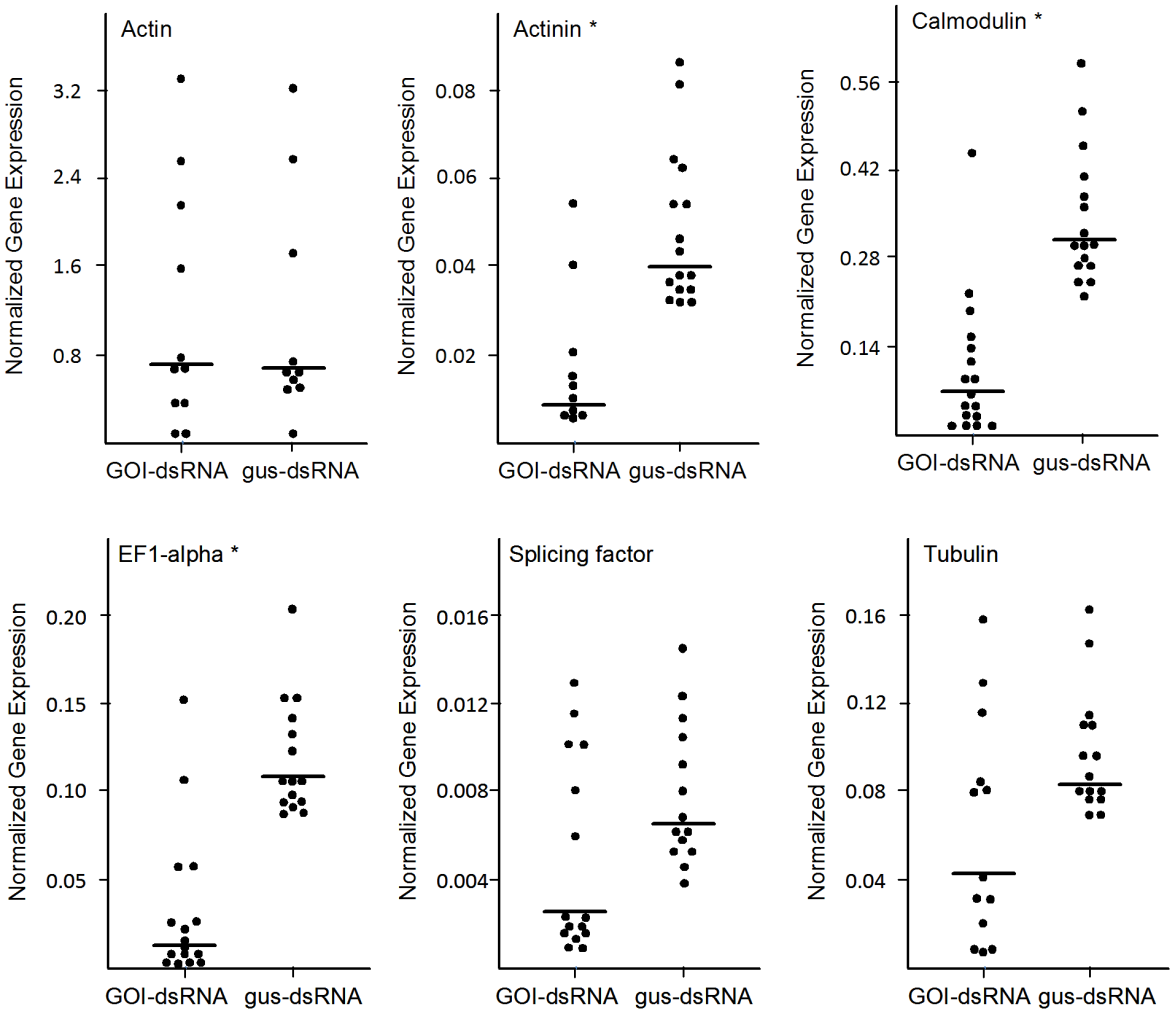
Scatter plots of the gene of interest (GOI) expression levels in young-of-the-year (YOY) treatments and *gus*-siRNA negative control individuals. The expression level of each gene of interest (GOI) is expressed relative to that of the ribosomal protein gene expression level for each individual lamprey. Each point represents the average of three replicate measurements from an individual larva, and the horizontal bar denotes the median gene expression level for the entire group treated. A P-value less than 0.05 (*) denotes that the median value of the level of transcripts in the GOI-siRNA-treated larvae is significantly different from that of the *gus*-siRNA control treatments, using a Mann-Whitney U test.

Delivering siRNAs to larvae might also be achieved by direct injection, but a primary aim of this study was to assess whether ingested siRNAs could enter gut cells and induce RNAi in lamprey larvae. An initial trial to feed naked (no transfection reagents) tubulin-specific siRNAs, at a concentration of 5 µg/ml, to YOY larvae failed to induce an RNAi response. Rather than use higher concentrations of siRNA, we opted to try using a liposome-based transfection reagent, Lipofectamine 2000 (Invitrogen), to improve siRNA delivery, as transfection reagents and nanoparticles have been used previously to deliver siRNAs to intestinal cells in mice by oral delivery [30],[31]. Exposure of the YOY (10–12 weeks old, 7–12 mm long) larvae to liposome- encapsulated siRNAs for a 6 h period proved effective in inducing RNAi in the mid-section tissues of the YOY larvae, 2 days post-treatment, for three of the six siRNAs tested (Figure 1). siRNAs targeting elongation factor, calmodulin, and α- actinin reduced the median level of transcripts 2.5, 3.6, and 5.0–fold, respectively, relative to the *gus*-siRNA-treated control animals (Mann-Whitney U test, P<0.05,). The other three siRNAs (actin, splicing factor, and tubulin siRNAs) showed no significant knockdown of transcripts, as there was a wide range of expression levels for these three genes in many of the animals, both in the negative controls and gene-specific siRNA treatments.

Following the 6 h exposure to the siRNAs, YOY larvae were observed for 8 days post-treatment to assess whether the siRNAs would induce mortality. The siRNA treatments were conducted in small, movement-restricting volumes of water, and unfortunately, each of the negative control treatments (water only, Lipofectamine only, or the gus-siRNA) sustained modest, but equivalent levels of mortality (23–28%; P>0.8, χ^2^ test, Yates correction) over this period. The fact that neither the gus-siRNA nor the Lipofectamine-alone treatments induced any higher mortality than the water-treated controls suggests that non-specific siRNAs and the transfection reagents used in our experiments were not particularly toxic to the larvae. While most of the siRNA treatments resulted in mortalities that were either equal or only slightly higher than this mortality rate, the tubulin siRNA treatment showed a significant increase in mortality relative to the negative control (Table 6), with 48% dead after the eight days (χ^2^ test, Yates correction, P<0.05). While the tubulin transcripts at day 2 post-treatment had not apparently been reduced significantly, the knockdown proved to be sufficient to induce mortality in a modest percentage of animals. It is worth noting that of the animals assessed for tubulin transcript levels (Figure 1), 50% of the animals showed between 50 and 87% reduction in the median levels of tubulin transcripts; if they were permitted to develop, they may represent those animals that succumbed to the lethal effects of the siRNA treatment.

**Table 6:** Percent mortality of young-of-the-year larvae 8 days after treatment with the siRNAs.

siRNA treatment	Percent mortality
Water only	23.3
Lipofectamine only	26.7
Gus (negative control)	28.3
α-actinin	30.5
Actin	29.0
Calmodulin	23.5
Elongation factor	30.0
Splicing factor	34.0
Tubulin	47.5 * (P<0.05)

The values represent the average mortality of two independent treatments of 15 individuals. Values were compared to the *gus*-siRNA negative controls using Chi-square analyses with Yates' correction for continuity and the only statistically different value is highlighted (*) and the associated P-value is provided in parentheses.

In contrast, for those siRNAs that had induced a significant reduction in the transcript levels (elongation factor, calmodulin, and α- actinin) in the YOY larvae, these animals did not suffer any higher mortality rates than the negative control animals (P>0.05, χ^2^ test, Yates correction), which suggests either that the expression of these genes recovered soon after day 2, and/or the knockdown of those transcripts was not critical to larval survival at that period of development.

**Figure 2:**
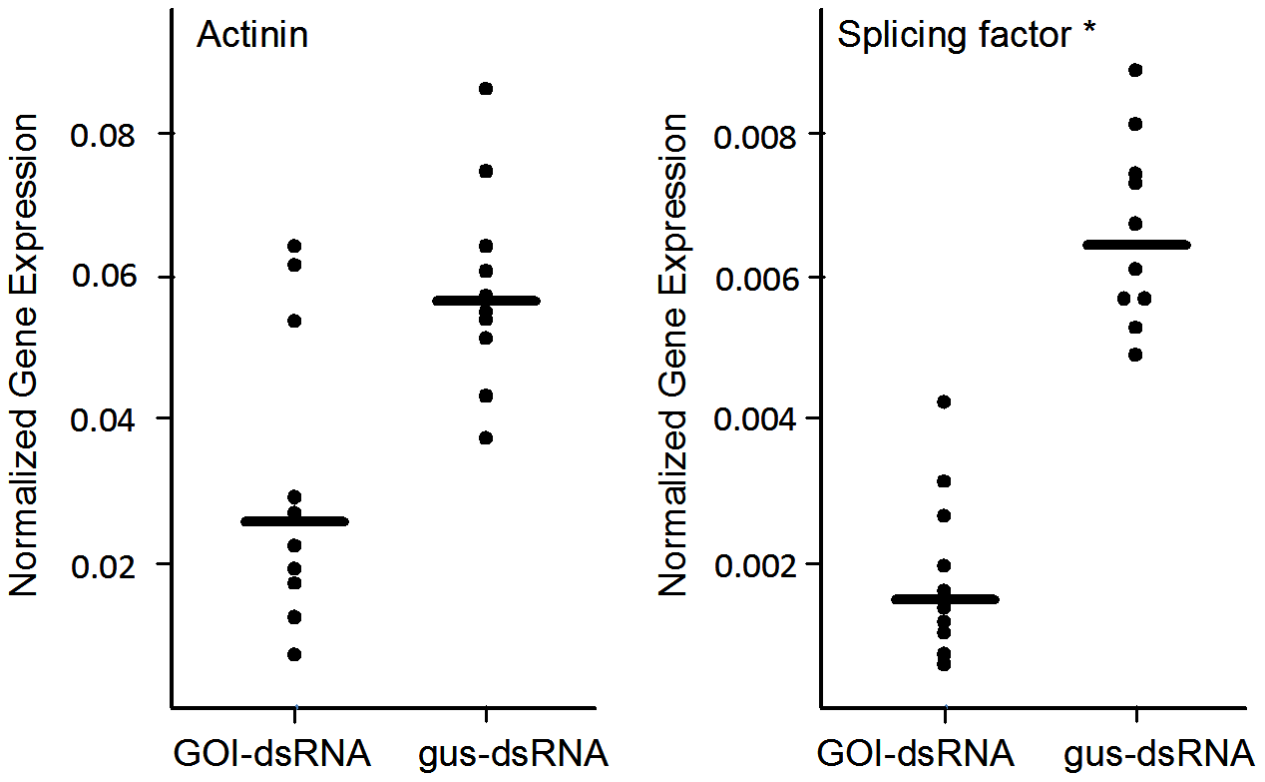
Scatter plots of transcript levels of two genes, α-actinin and splicing factor, in older larvae (1+ years) following exposure to siRNAs. The expression level of each gene of interest (GOI) is expressed relative to that of the ribosomal protein gene expression level for each individual lamprey. Each point represents the average of three replicate measurements from a single larva's gut, and the horizontal bar denotes the median gene expression level for the entire group treated. A P-value less than 0.05 (*) denotes that the median value of the level of transcripts in the GOI-siRNA-treated larvae is significantly different from that of the *gus*-siRNA control treatments, using a Mann-Whitney U test.

Older larvae, ranging in age between 1 and 2 years old and ranging in size between 40 and 80 mm, were treated with two of the siRNAs (α-actinin and splicing factor siRNAs) at the same concentration used in the YOY treatments, and the splicing factor siRNA induced a significant 70% knockdown in transcript levels (P<0.05, Mann-Whitney U test), whereas the α-actinin transcript levels were variable and showed no significant knockdown (Figure 2). No siRNA-induced mortality was observed in these larger animals.

### Species-specificity of the siRNAs?

The same PCR primers that were used to isolate the target gene fragments from sea lamprey cDNA were used to isolate putative homologues for five of the six genes in northern brook lampreys. The only gene for which no northern brook lamprey sequence could be obtained was the actinin gene. Although only small portions of the five remaining genes (ranging between 251 and 353 bp) could be compared, none of the gene fragments showed more than 3.2% divergence from each other (Table 7), and in all genes examined, there was no 19 to 21 nt stretch that was unique to sea lampreys. All siRNAs used to induce RNAi in sea lampreys matched the northern brook lamprey sequences between 90 and 100%. Although not experimentally determined, this degree of identity of the gene sequences in the two species should permit the siRNAs used in this study to anneal to the northern brook lamprey mRNA and to elicit the same RNAi effects.

**Table 7:** Nucleotide sequence identities between the gene fragments isolated from sea lamprey *P. marinus* (*P.m*.) and northern brook lamprey *I. fossor* (*I.f*.).

Gene	Fragment length (bp)	% identity between *P.m.* and *I.f*. sequences	Potential species-specific siRNA?
Actin	353	98.9	No
Actinin	289	N.D.	?
Calmodulin	119	100	No
Elongation factor	332	99.7	No
Splicing factor	251	96.8	No
Tubulin	269	99.1	No

A search for potential species-specific siRNAs was conducted by looking for stretches of 21 contiguous nucleotides with at least 3 nucleotide differences between the two sequences; none were found.

## Discussion

RNAi has been widely used as a molecular biology tool to identify gene function in many eukaryotic organisms [32],[33], but it also has enormous potential to be applied in the treatment of human diseases [34] and in the control of pest organisms [21],[35]. In this study, siRNAs were delivered to both sea lamprey embryos and larvae and resulted in substantial knockdown of transcript levels of several of the siRNA-targeted genes. This is the first demonstration of siRNA-mediated knockdown in this basal vertebrate. Indeed, there have been only a handful of studies that have achieved down-regulation of gene expression in lampreys, all of which have used injected synthetic morpholinos to block translation of the targeted genes in embryos [25],[36]–[38]. Morpholinos have been routinely used by fish biologists to study gene functions [39], and siRNAs have been largely ignored for use in fish [40]. Early attempts to induce RNAi in fish used long dsRNA molecules [41]–[43], which were subsequently recognized as inducers of an interferon-mediated, non-specific reduction in total gene expression [44]. With that realization, fish biologists switched to the use of siRNAs, but even those early trials observed inconsistent results when injecting fish embryos, ranging from only small reductions in gene expression [45] to unexpected abnormal morphological defects and embryonic death [46]. It has been noted that fish embryo developmental defects can occur if high concentrations of siRNAs are used, as the siRNAs can interfere with the cell's microRNA processing pathway [47]. As a consequence of these mixed successes, progress in RNAi research in fish has been slow [48]. In our study, we observed only a small degree of embryonic mortality following siRNA injections, suggesting that lamprey embryos may be more amenable to siRNA-mediated RNAi than some other fish. Our feeding method of delivery of siRNAs may also prove useful for other fish species, as a means of avoiding the lethal effects of siRNA delivery to embryos. Regardless of its utility in other fish species, it will certainly provide a valuable gene validation tool in lampreys, as well as its possible use in the development of novel lamprey control strategies.

An important factor affecting RNAi efficiency is the delivery of the siRNA to the target cells. Our demonstration that siRNAs fed to lamprey larvae can induce RNAi and even kill the larvae following knockdown of the targeted gene is the first example of oral delivery of siRNAs in a non-mammalian vertebrate. Oral delivery of siRNAs in vertebrates has also been achieved in mice, as models for possible use as human therapeutics; for example, siRNAs encased in glucan microspheres [30] or bound to thioketal nanoparticles [31] successfully induced RNAi in intestinal cells of mice when the formulations were administered by oral gavage. Like the mice studies, we only observed RNAi when the siRNAs were delivered with a transfection reagent. Given the harsh, digestive environment of the gut, it is not surprising that unprotected siRNAs were not effective. A range of transfection reagents and nanoparticles have been developed for the delivery of medicines to humans, and are now being considered for oral delivery of therapeutic siRNAs to humans [49],[50]. As we did not observe complete knockdown of any the genes targeted in the lampreys, it would be worthwhile determining whether any of these other microcarriers could deliver siRNAs more effectively to the lamprey gut cells. For each of the four effective siRNAs (α-actinin, calmodulin, splicing factor and tubulin), the target genes' expressions were only partially reduced in the embryos, ranging between 49 and 86%. RNAi efficiency could potentially be enhanced by increasing the dose of siRNA [21],[33], but seldom does RNAi fully eliminate all target transcripts [51]. Greater than 90% gene silencing can be attained in cell cultures [52], where it is easy to deliver large doses of siRNAs to the cells. Injection of the siRNAs into embryos can potentially deliver a large dose, but nevertheless, the siRNA may still not be fully effective, as the efficacy of any particular siRNA to produce a perceptible RNAi phenotype is influenced by a variety of factors, including the transcription rate of the targeted gene, the overall stability of the mRNA and its encoded protein, and the efficiency of each siRNA to bind to its target [53] All of the genes targeted in this study are housekeeping genes, and while full loss of function of these critical genes was expected to be lethal, incomplete knockdown of a target gene may weaken but not necessarily kill the cell or organism. Nevertheless, partial knockdown of three of the siRNA-targeted genes was sufficient to induce mortality in the developing embryos, which suggests that for those genes at least, partial loss of gene expression can have severe consequences in these embryos.

The knockdown of three target genes in the YOY larvae and one gene in the 1 yr+ larvae is a significant finding as it not only indicates that ingested siRNAs can enter lamprey cells and induce RNAi, but that the siRNAs have potential to kill larvae of various ages if RNAi can be sustained over a sufficiently long exposure period. In this study, larvae were exposed to the siRNAs for only six hours, but if feeding formulations can be developed that allow the slower release of particulates that contain siRNAs, then it may be possible to deliver lethal doses of the siRNAs to the filter-feeding lamprey larvae over a longer period. Chitosan nanoparticles have been used to orally-deliver dsRNAs to shrimp [54] and mosquito larvae [55], and may prove effective for the stable encapsulation of lampricidal siRNAs in feeding formulations that could be distributed into local streams to control the pest animals. If siRNAs are to be used as lampricides, they will need to be produced in large quantities. They can already be produced chemically, but other simple and cost-effective methods of siRNA production have also been developed, including a readily-scalable, *in vitro* transcription method [56] and a microbial-expression system [57], where bacteria were used to produce siRNAs that were then mixed into food pellets and fed to shrimp [54].

The primary rationale for considering siRNAs as possible lampricides is that they offer the possibility of being species-specific. Previous studies have demonstrated that even for highly conserved genes, it may be possible to identify short (21 nt) sequences that are unique to a target species [22],[24]. In this study, we chose to target highly conserved housekeeping genes, only because the lamprey genome was not fully annotated when this study commenced, and finding well-conserved genes within the database would be more straightforward. None of these genes would be ideal as targets for a lampricide however, as there is a high probability that some other species would have highly similar if not identical sequences to any siRNA used to target these genes. In fact, we found that the same primers used to amplify the six genes from sea lamprey cDNA could be used to amplify nearly identical sequences from the northern brook lamprey, a native species within the Great Lakes watershed. A comparison of the sea lamprey siRNA sequences with the corresponding genes in northern brook lampreys showed little or no differences, which suggests that the siRNAs tested in this study would, in all likelihood, cross-react with the northern brook lamprey genes.

The siRNAs developed and tested for this study nevertheless served as an important proof-of-concept test of the technology, and this study proved two important points: 1) it is possible to induce RNAi in lampreys that feed on siRNAs; and 2) knockdown of some genes in lamprey embryos and larvae can induce mortality. To develop the technology to the point of being an effective lampricide will require further developments in producing large quantities of siRNAs, developing effective stream distribution formulations, and improving our knowledge of lamprey genes to design sea lamprey-specific siRNAs that selectively kill the pest but leave other species unharmed.
